# Melanocytic matricoma: a pigmented lesion on the forehead^☆^

**DOI:** 10.1016/j.abd.2023.05.011

**Published:** 2024-06-03

**Authors:** Teresa Alonso-de-León, Carlos Barrera-Ochoa, Luis Enrique Cano-Aguilar, Katia Lizette Munguia-Galeano, Jorge Felipe Flores-Ochoa, María Elisa Vega-Memije

**Affiliations:** a Department of Dermatology, General Hospital “Dr. Manuel Gea González”, Mexico City, Mexico; b Department of Pathology, “Instituto Nacional de Rehabilitación”, Mexico City, Mexico; c Department of Internal Medicine, Mexican Social Security Institute, Mexico City, Mexico

*Dear Editor,*

A 74-year-old woman presented to our outpatient dermatology clinic with a 4-year history of an asymptomatic, pigmented tumor located on the forehead. On physical examination, we found a 5 mm, dark brown colored papule, surrounded by an erythematous rim. Past medical history was unremarkable. On dermoscopy, we found blue-gray ovoid nests, ulceration and peripheral telangiectasias (Fig. 1). The tumor was clinically diagnosed as pigmented basal cell carcinoma. A cutaneous biopsy was performed, and the histopathologic study revealed an epithelial, well-circumscribed neoformation that was composed of basophilic cells with hyperchromatic nuclei, a scarce cytoplasm, and prominent nucleoli. Mixed with these basaloid cells, there were multiple cells with basophilic nuclei and eosinophilic cytoplasm that were arranged in small nests. Sparse ghost cells were also found. There were multiple dendritic and pigmented melanocytes as well as areas of compacted keratinization (Fig. 2). Immunohistochemical study with BerEP4 turned positive in basaloid areas. Melanocytic matricoma diagnosis was concluded and a complete tumor resection was performed. The patient remained clinically disease-free during follow-up consultation.

**Figure 1:**
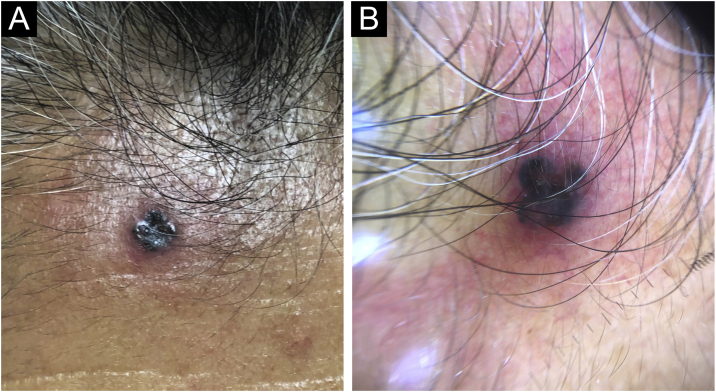
Physical examination. (A) A dark brown-black colored papule. (B) Dermoscopy. Blue-gray ovoid nests and ulceration, surrounded by a 3 mm erythematous and elevated rim.

**Figure 2:**
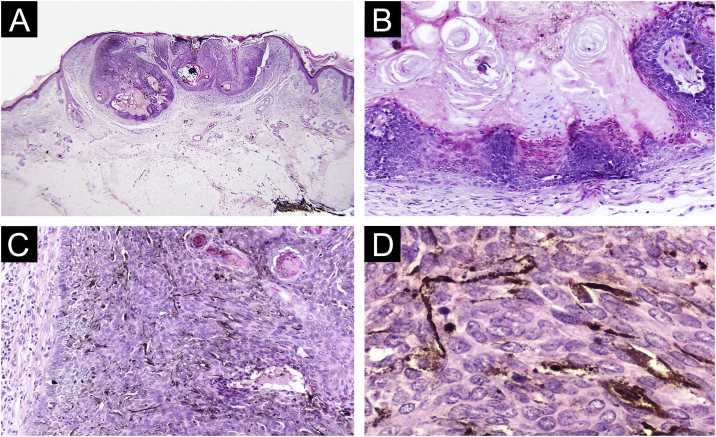
Histopathology findings (A) Well circumscribed tumor arranged in lobes (Hematoxylin & eosin, 4×). (B) Basaloid and ghost cells with compact keratinization (Hematoxylin & eosin, 10×). (C) Basaloid cells and numerous dendritic melanocytes (Hematoxylin & eosin, 20×). (D) Dendritic pigmented melanocytes (Hematoxylin & eosin, 40×).

Melanocytic matricoma is considered an adnexal tumor with matrical differentiation.^1^, ^2^ This newly described neoformation predominates in males, and it is strongly associated with sun-damaged skin in elderly patients, with a mean age of 71 years at presentation.^3^, ^4^ Only 32 cases have been reported in international literature. This uncommon tumor is predominantly located on the head, particularly in the nose and preauricular area, but it has also been reported on the neck, trunk, and extremities.^4^ Its classical clinical presentation is described as a small, well-circumscribed, nodular tumor with an asymmetric dark pigmentation.^1^, ^4^ Melanocytic matricoma is a biphasic tumor that comprises an epithelial component with matrical differentiation, and a melanocytic component with dendritic melanocytes. The epithelial component consists of basaloid cells with scarce cytoplasm, round nuclei, dotted nuclear chromatin, and prominent nucleoli. These basaloid cells might acquire mild to moderate pleomorphism with a slightly elevated mitotic activity, thus denominated matrical and supramatrical cells. These cells show an abrupt or gradual transition to ghost cells. In contrast, the melanocytic component appears as a well-circumscribed arranged nodule that is composed of melanocytes, intermixed with matrical and supramatrical cells, as well as foci of ghost cells. The epithelial component shows positivity for cytokeratin and beta-catenin, whereas dendritic melanocytes are highlighted by HMB-45, S-100, and Melan-A.^5^ Most tumors involve superficial to deeper dermis, without an evident epidermal or adnexal connection.^1^ The histopathologic and immunohistochemical findings suggest that melanocytic matricoma resembles anagen hair growth. Therefore, melanocytic matricoma is currently classified as a cutaneous adnexal tumor with both follicular and matrical differentiation.^4^

Clinical differential diagnosis includes basal cell carcinoma, melanoma, and hemangioma, but the main clinical differential diagnosis is pilomatrixoma^1^ (Table 1).^6^, ^7^, ^8^ This benign cutaneous tumor is found predominantly in young females (average 20 years), localized frequently on the neck and extremities, and it is clinically presented as a multilobulated and firm subcutaneous nodule. In contrast, histopathologic differential diagnosis includes tumors with matrical differentiation, such as pilomatrixoma, pigmented pilomatrixoma, and basal cell carcinoma with matrical differentiation (Table 2).^9^

**Table 1:** Differential diagnosis of melanocytic matricoma.^4^, ^6^.

	Melanocytic Matricoma	Pilomatrixoma	Basal Cell Carcinoma	Melanoma
**Common age group**	Elderly individuals	Children, younger than 10 years.	Adults, elderly	Young and middle-aged individuals
**Sex**	M > F	F > M	M > F	F > M
**Size (cm)**	0.2 ‒ 1.5	0.5 ‒ 3	0.5 ‒ 10	Variable
**Clinical Features**	Pigmented nodule, polypoid or exophytic, rarely ulcerated.	Solitary, asymptomatic, slow growing, cystic, or firm nodule.	Slow growing, ulcerated nodule or plaque.	Asymmetric macule or nodule with irregular borders, might present variations in color within the lesion
**Sun Damaged Skin**	Significant	Not significant	Significant	Not significant
**Dermoscopy**	Homogeneous blue or patched pigmentation. Fork-shaped glasses	Blue pigmentation, dilated vessels.	Large gray-blue ovoid nests, multiple blue-gray globules, maple leaf-like areas	Atypical network, blue whitish veil, atypical vascular pattern, irregular globules, irregular streaks, regression structures
**Site**	Head and neck, upper extremities.	Head and neck, upper extremities.	Head and neck, upper extremities (arms and hands).	Trunk, limbs, acral regions, head

**Table 2:** Histopathological differences between melanocytic matricoma, pigmented pilomatrixoma and pigmented basal cell carcinoma with matrical differentiation.^4^, ^9^, ^10^

	Pigmented Pilomatrixoma	Melanocytic Matricoma	Pigmented Basal Cell Carcinoma with Matrical Differentiation
**Epidermis involvement**	Any	Atrophic, or hyperkeratotic with acanthosis	Any
**Histopathological pattern**	Nodular or multinodular with cystic areas	Single solid well-defined nodule, usually no cystic areas	Nodular pattern accompanied by either superficial pattern, cystic change or an infiltrative pattern
**Location in the dermis**	In deep dermis with frequent extension to adipose tissue	In superficial to medium reticular dermis	Dermis to subcutaneous tissue in some cases
**Cell types**	Variable mixture of basaloid, transitional and ghost cells	Matrix and supra-matrix cells, few occasional ghost cells	Basaloid follicular and germinative cells, ghost cells with basaloid appearing matrical cells in periphery
**Keratinization Type**	Pilar and sometimes infundibular keratinization	Pilar keratinization	Central abrupt matrical keratinization
**Histopathological alterations of the dermis**	Connective tissue: Blood vessels, infiltrate of mixed inflammatory cells, giant foreign body cells.	Sclerotic stromal response, actinic elastosis	Actinic elastosis
	Sometimes: hemosiderin, melanin, bone and, rarely, amyloid deposition.		
**Calcium deposition**	In 80% of cases	Infrequent	Presented in some cases
**Foreign body reaction**	Very frequent	Infrequent	Infrequent
**Ghost cells**	In the center of the tumor, almost always present	Present in small foci mixed with pigmented, dendritic melanocytes	Present in most of the tumor
**Mitosis**	There may be cytological atypia and mitotic figures	There may be cytological atypia and mitotic figures	Present cytologic atypia and high mitotic figures
**Melanin**	Melanin is rare, some dendritic melanocytes	Pigmented, inside melanophages, and dentritic melanocytes distributed asymmetrically.	Tumor cells with melanin in cytoplasm. No prominence of intra tumoral melanocytes

The importance of recognizing this recently described tumor is based on its unknown prognosis and lack of treatment options, besides surgery. Therefore, it is important to consider melanocytic matricoma in elderly, sun-damaged skin patients with a newly discovered pigmented neoplasm, besides pigmented basal cell carcinoma and melanoma, requiring wider surgical margins and a closer follow-up. It is essential to report all melanocytic matricoma cases to establish its clinical course and prognostic features.

## Financial support

None declared.

## Authors’ contributions

Teresa Alonso-de-León: Writing of the manuscript or critical review of important intellectual content; data collection, analysis, and interpretation; effective participation in the research guidance; critical review of the literature; final approval of the final version of the manuscript.

Carlos Barrera-Ochoa: Critical review of the literature; data collection; analysis and interpretation.

Luis Enrique Cano-Aguilar: Writing of the manuscript or critical review of important intellectual content; data collection, analysis and interpretation; effective participation in the research guidance; intellectual participation in the propaedeutic and/or therapeutic conduct of the studied cases; critical review of the literature.

Katia Lizette Munguia-Galeano: Critical review of the literature.

Jorge Felipe Flores-Ochoa: Critical review of the literature.

Maria Elisa Vega-Memije: Effective participation in the research guidance; intellectual participation in the propaedeutic and/or therapeutic conduct of the studied cases; critical review of the literature.

## Conflicts of interest

None declared.
